# Prescription of off-label and unlicensed drugs for preterm infants in a neonatal intensive care unit

**DOI:** 10.5935/0103-507X.20210034

**Published:** 2021

**Authors:** Verônica Cheles Vieira, Renart Santos Costa, Raquel Cristina Gomes Lima, Daiane Borges Queiroz, Danielle Souto de Medeiros

**Affiliations:** 1 Instituto Multidisciplinar em Saúde, Universidade Federal da Bahia - Vitória da Conquista (BA), Brazil.

**Keywords:** Infant, preterm, Infant, newborn, Intensive care units, neonatal, Drug prescription, Off-label use, Recém-nascido prematuro, Recém-nascido, Unidades de terapia intensiva neonatal, Prescrição de medicamentos, Uso *off-label*

## Abstract

**Objective:**

To evaluate the use of off-label and unlicensed medications in preterm infants hospitalized in a neonatal intensive care unit.

**Methods:**

This nonconcurrent cohort study included preterm infants admitted to 3 neonatal intensive care units in 2016 and 2017 who were followed up during the neonatal period. The type and number of medications used were recorded for the entire period and classified based on the Anatomical Therapeutic Chemical. Descriptive and bivariate data analyses were performed to assess associations between the number of drugs used (total, off-label and unlicensed) and the explanatory variables of interest.

**Results:**

Four hundred preterm infants received 16,143 prescriptions for 86 different pharmaceuticals; 51.9% of these medications were classified as off-label and 23.5% as unlicensed. The most prescribed drugs were gentamicin and ampicillin (17.5% and 15.5% among off-label, respectively) and caffeine (75.5% among unlicensed). The results indicated significant associations between the use of off-label drugs and lower gestational age, low birth weight, lower 5-minute Apgar score, advanced resuscitation maneuver in the delivery room and death. The prescription of unlicensed drugs was associated with lower gestational age, low birth weight and 5-minute Apgar score below 7.

**Conclusion:**

Neonates admitted to neonatal intensive care units are highly exposed to off-label and unlicensed medications. Further studies are needed to achieve greater safety and quality of drug therapy used in neonatology.

## Abstract (pt)

**Objetivo:**

Avaliar o uso de medicamentos *off-label* e sem licença em recém-nascidos prematuros hospitalizados em unidade de terapia intensiva neonatal.

**Métodos:**

Estudo de coorte não concorrente, incluindo prematuros admitidos em três unidades de terapia intensiva neonatais, nos anos de 2016 e 2017, acompanhados durante o período neonatal. O uso de medicamentos e o número foram registrados para todo o período e classificados segundo a *Anatomical Therapeutic Chemical*. Foram realizadas análises descritivas e bivariadas dos dados para avaliar associações entre o número de medicamentos utilizados (total, *off-label* e sem licença) e as variáveis explicativas de interesse.

**Resultados:**

Os 400 neonatos prematuros utilizaram 16.143 medicamentos, com 86 especialidades diferentes; 51,9% desses itens foram classificados como *off-label* e 23,5% como sem licença. Os mais prescritos foram gentamicina e ampicilina (17,5% e 15,5% dos *off-label*, respectivamente) e cafeína (75,5% dos não licenciados). O estudo demonstrou associações significativas do uso de medicamentos *off-label* com a menor idade gestacional, baixo peso ao nascer, menor escore de Apgar no quinto minuto, manobra de reanimação avançada em sala de parto e óbito. Com os medicamentos não licenciados, foram verificadas associações com a menor idade gestacional, baixo peso ao nascer e escore de Apgar no quinto minuto menor que 7.

**Conclusão:**

Os neonatos internados em unidades de terapia intensiva neonatais são muito expostos ao uso de medicamentos *off-label* e sem licença. Tornam-se necessários mais investimentos em estudos para alcançar maior segurança e qualidade da terapêutica medicamentosa empregada em neonatologia.

## INTRODUCTION

The use of medications is essential in health care due to their curative, prophylactic or palliative effects; however, medications can also pose risks to patient health. When medications are prescribed, their quality, cost-effectiveness and safety should be considered. Thus, clinical trials are necessary for safe prescriptions, especially among high-risk groups such as pregnant women, the elderly and children.^(1,2)^

Pediatric patients, especially preterm neonates, are a vulnerable group, especially when they require intensive care due to an increased need for pharmacological treatments. Drug metabolism and renal function are immature in these patients, and there are differences in the sensitivity of target organs. Thus, the half-life of a drug tends to be longer, and accumulation in the body may occur.^(3,4)^

In Brazil, the National Health Surveillance Agency (Agência Nacional de Vigilância Sanitária - ANVISA) is the agency responsible for authorizing drug registrations in Brazil based on data and information from nationally recognized regulatory agencies. However, there is currently no specific regulation for drugs intended for the pediatric population. Because of ethical dilemmas, the field of pediatrics faces substantial challenges with regard to conducting clinical trials involving this population.^(3)^

In the care of newborns, the scarcity of medication studies is so substantial that many experts even consider this specialty almost “experimental” because 40% to 80% of the drugs used in neonatal intensive care units (ICUs) are off-label or unlicensed.^(5,6)^

Off-label medication refers to a drug that is used for a different purpose than that described on its label or package insert in regard to indication, route of administration, dosage and age group.^(7)^ These medications, however, are approved by regulatory agencies for use.^(8)^

Unlicensed drugs are not approved for use because they do not have a specific dosage and are contraindicated for specific populations and are modified, such as the preparation and use of extemporaneous formulations conceived from existing pharmaceutical formulations through the grinding of tablets, dilution of oral liquids or opening of capsules.^(8,9)^

The prescription of these drugs has resulted in important legal and clinical debates because studies associate them with an increased risk of exposure to harmful or potentially harmful excipients, both in adults and in the pediatric population.^(10,11)^

Considering the risks of this practice, the aim of this study is to evaluate the use of off-label and unlicensed drugs in preterm newborns hospitalized in neonatal ICUs.

## METHODS

This is a nonconcurrent, hospital-based cohort study that is part of a broader study titled “Preterm birth cohort - survival and morbidity in preterm infants in neonatal intensive care units in the municipality of Vitória da Conquista - BA: a nonconcurrent cohort study”.

The study was conducted in Vitória da Conquista (BA). Preterm infants admitted to 3 neonatal ICUs of 3 hospitals (2 public and 1 private) were included. Each neonatal ICU offered 10 beds and had a trained multidisciplinary team for the care of high-risk newborns. The neonatal ICU units included in the study served as training sites for the Medical Residency Program in the Specialization of Pediatrics and Neonatology and had similar clinical practice protocols.

The study included all preterm infants admitted to the neonatal ICU of 3 hospitals in the municipality of Vitória da Conquista between January 1^st^, 2016, and December 31^st^, 2017. The population was monitored from the day of admission during the neonatal period, respecting censorship of 27 days (interruption of follow-up). Data were collected by analyzing the medical records of preterm infants; the records were stored in the Medical Records and Statistics Service (MRSS) of the hospitals.

The exclusion criterion was major congenital anomaly (severe anatomical changes; for this study, patients with the following conditions were excluded: complex congenital heart diseases, gastrointestinal tract atresias, abdominal wall defects, hydrocephalus, encephalocele and diaphragmatic hernia).

The sample was obtained by convenience (n = 400). However, the smallest sample size needed to represent the preterm population of the region was estimated at 384, considering the following parameters: infinite population size (given that it is not possible to estimate the total number of preterm infants who would require intensive neonatal care because the region contains many municipalities), expected frequency of 50% (considering the multiple outcomes evaluated), 5% accuracy and 95% confidence interval.

The instrument used to perform the collections was a questionnaire adapted from the national survey on delivery and birth in Brazil.^(12)^ Voluntary health researchers, under the supervision of neonatologists, were responsible for data collection using a digital questionnaire uploaded in tablets with KoBo Toolbox software, version 1.4.8. A pilot study was conducted in May 2018 using medical records of preterm infants hospitalized in the period prior to the study. The main data recovery occurred from June 2018 to May 2019.

The dependent variables of this study included the use (categorized as yes or no) and number of medications related to the length of stay in the neonatal ICU. Each pharmaceutical specialty was registered by its generic name, pharmaceutical form and route of administration. Pharmacological classification was performed based on the Anatomical Therapeutic Chemical (ATC) classification recommended by the World Health Organization (WHO).^(13)^ For the present study, drug classifications relative to levels 1 (anatomical) and 2 (therapeutic) were used. Level 5 (chemical) was also used to describe the most commonly used products.

The drugs were also classified as off-label and unlicensed for the population in accordance with the American criteria, based on Costa,^(14)^ and through the international database Drug Dex-Micromedex^®^ (https://www.micromedexsolutions.com/home/dispatch).^(15)^ Off-label drugs were defined as those for which age, indication or route of administration differed from that authorized by the health agency, in this case the United States Food and Drug Administration (FDA).

The following independent variables were used: gestational age (moderate or late, very preterm or extremely preterm), birth weight (≥ 2,500g, ≥ 1,500g and < 2,500g, ≥ 1,000g and < 1,500g or < 1,000g), 5-minute Apgar score (≥ 7 or < 7), resuscitation in the delivery room (did not perform, positive-pressure ventilation or advanced resuscitation - positive-pressure ventilation plus cardiac massage and/or drugs) and death (yes or no).

Descriptive analyses were performed using a simple frequency distribution. The prevalence of drug use (total, off-label and unlicensed) was calculated from the number of preterm infants who had a record of use of at least 1 drug during their stay in the neonatal ICU, divided by the total number of preterm infants.

Bivariate analysis was performed to evaluate associations between the number of drugs used (total, off-label and unlicensed) and the explanatory variables of interest. For this purpose, continuous variables were described based on each explanatory variable using the median and the maximum and minimum values. The differences between the selected variables were assessed by nonparametric methods: the Mann-Whitney-Wilcoxon test for variables with 2 categories and the Kruskal-Wallis test for those with more than 2 categories, with a significance level lower than 5%. For all variables, the number of drugs used was described based on each categorization (total, off-label and unlicensed) using boxplot graphs. Stata software, version 15.0 (Stata Corporation, College Station, USA), was used for data analysis.

All phases of this study were performed in accordance with ethical and legal guidelines detailed in resolution 466/12 of the National Health Council of the Ministry of Health (Conselho Nacional de Saúde do Ministério da Saúde). The study titled “Survival and morbidity in preterm infants in neonatal intensive care units in the municipality of Vitória da Conquista - BA: a nonconcurrent cohort study” was approved by the Human Research Ethics Committee of the *Instituto Multidisciplinar em Saúde* of the *Universidade Federal da Bahia* (UFBA; CAAE: 79450717.4.0000.5556) on February 5th, 2018.

## RESULTS

During the study period, a total of 592 preterm infants were admitted to the neonatal ICU units, of whom, 37 were excluded because they had major congenital malformations, and 155 were excluded because of missing or incomplete records, leaving 400 preterm infants who were included in the study.

Among the participants, the gestational age ranged from 23^0/7^ to 36^6/7^ weeks, with 59.3% being moderate or late preterm, 29.2% being very preterm and 11.5% being extreme preterm. More than half of the participants had a weight greater than or equal to 1,500g (67.0%), had a 5-minute Apgar score of 7 or higher (86.0%) and did not require resuscitation in the delivery room (54.3%), while 16.0% died during hospitalization (Table 1).

**Table 1:** Characteristics of the studied population

Variables	n (%)
Gestational age	
Moderate/late preterm	237 (59.3)
Very preterm	117 (29.2)
Extremely preterm	46 (11.5)
Birth weight (g)	
≥ 2,500	48 (12.0)
≥ 1,500 and < 2,500	178 (45.0)
≥ 1,000 and < 1,500	122 (30.0)
< 1,000	52 (13.0)
5-minute Apgar score	
≥ 7	335 (86.0)
< 7	54 (14.0)
Resuscitation maneuver in the delivery room	
No	217 (54.3)
Only PPV	156 (39.0)
Advanced maneuvers	27 (6.7)
Death	
Yes	64 (16.0)
No	336 (84.0)

PPV - positive-pressure ventilation

The prevalence of prescribed medications in the population was 89.8% (95% confidence interval - 95%CI 86.3 - 92.4%). Regarding the use of at least 1 off-label product, the prevalence was 79.0% (95%CI 74.7 - 82.7%). Unlicensed medications were prescribed to 55.5% (95%CI 50.5 - 60.3%) of preterm infants.

The number of medications prescribed per patient ranged from zero to 217, with a median of 30. For off-label medications, number of medications prescribed per patient ranged from zero to 161, with a median of 13, and for unlicensed medications, it ranged from zero to 56, with a median of 4.

Overall, the preterm infants included in this study received 16,143 prescriptions for 86 different pharmaceuticals. Among these prescriptions, 8,372 (51.9%) were for products classified as off-label, and 3,790 (23.5%) were classified as unlicensed. The most used pharmaceutical classes were systemic anti-infectives (36.9%) and drugs that acted on the nervous system (27.6%) and on the digestive system and metabolism (24.5%). Among the off-label medications, systemic anti-infectives predominated (63.8%), especially those of the therapeutic subgroup systemic antibacterials (56.0%). Most unlicensed products acted on the nervous system (91.5%), mainly in the psychoanalytic subgroup (75.4%) (Table 2).

**Table 2:** Distribution of pharmaceutical classes used by preterm neonates, based on anatomical and therapeutic classification (Anatomical Therapeutic Chemical levels 1 and 2)

Anatomical and therapeutic group	Total	Off-label	Unlicensed
Digestive system and metabolism	3,961 (24.5)	975 (11.6)	
Medicines for acidogastric disorders	380 (2.3)	380 (4.5)	
Medicines for gastrointestinal disorders	442 (2.8)	442 (5.3)	
Medicines for biliary tract and liver	39 (0.2)	39 (0.4)	
Vitamins	2,959 (18.3)	114 (1.4)	
Blood and hematopoietic organs	327 (2.0)		212 (5.6)
Antianemic drugs	212 (1.3)		212 (5.6)
Cardiovascular system	953 (5.9)	731 (8.7)	83 (2.2)
Medications for cardiac disorders	650 (4.0)	509 (6.1)	2 (0.1)
Diuretic drugs	219 (1.3)	138 (1.6)	81 (2.1)
Vasoprotective drugs	79 (0.5)	79 (0.9)	
Drugs that act on the renin-angiotensin system	5 (0.1)	5 (0.1)	
Dermatological	38 (0.2)		
Genitourinary system and sex hormones	75 (0.5)	75 (0.9)	
Medicines for urological disorders	75 (0.5)	75 (0.9)	
Anti-infectives for systemic use	5,954 (36.9)	5,345 (63.8)	14 (0.4)
Antibacterials for systemic use	5,253 (32.5)	4,691 (56.0)	14 (0.4)
Antimycotics for systemic use	684 (4.2)	646 (7.7)	
Immunological sera and immunoglobulins	17 (0.1)	8 (0.1)	
Antineoplastic and immunomodulatory agents	11 (0.1)	11 (0.1)	
Antineoplastic agents	11 (0.1)	11 (0.1)	
Musculoskeletal system	30 (0.2)	23 (0.3)	
Anti-inflammatory and antirheumatic drugs	23 (0.1)	23 (0.3)	
Nervous system	4,451 (27.6)	983 (11.7)	3,468 (91.5)
Anesthetics	663 (4.1)	663 (7.9)	
Analgesics	68 (0.4)	55 (0.6)	13 (0.3)
Antiepileptics	634 (3.9)	41 (0.5)	593 (15.5)
Psychological	115 (0.7)	115 (1.4)	
Psychoanalytic	2,862 (17.7)		2,862 (75.4)
Other	109 (0.7)	109 (1.3)	
Antiparasitic products, insecticides and repellents	13 (0.1)		13 (0.3)
Antiprotozoa	13 (0.1)		13 (0.3)
Respiratory system	329 (2.0)	229 (2.7)	
Nasal preparations	69 (0.4)	69 (0.8)	
Medicines for obstructive diseases	161 (0.9)	160 (1.9)	
Sensory organs	1 (0.01)		
Total	16,143 (100.0)	8,372 (100.0)	3,790 (100.0)

Results expressed as n (%).

Considering the fifth level of the ATC classification, the most prescribed off-label drugs were gentamicin (17.5%), followed by ampicillin (15.5%) and amikacin (8.9%). Among the unlicensed drugs, the most prescribed were caffeine (75.5%), phenobarbital (15.6%) and ferrous sulfate (5.6%) (Table 3).

**Table 3:** Distribution of the most prescribed off-label and unlicensed drugs in decreasing order of use

Medications	ATC Code	Off-label	Unlicensed
Caffeine	N06BC01		2,862 (75.5)
Gentamicin	J01GB03	1,467 (17.5)	
Ampicillin	J01CA01	1,295 (15.5)	
Amikacin	J01GB06	741 (8.9)	
Fentanyl	N01AH01	656 (7.8)	
Fluconazole	J02AC01	642 (7.7)	
Phenobarbital	N03AA02		593 (15.6)
Cefepime	J01DE01	566 (6.8)	
Domperidone	A03FA03	412 (4.9)	
Meropenem	J01DH02	373 (4.5)	
Ranitidine	A02BA02	359 (4.3)	
Ferrous sulfate	B03AA07		212 (5.6)
Aminophylline	R03DA05	150 (1.8)	
Furosemide	C03CA01	138 (1.6)	
Vancomycin	J01XA01	125 (1.5)	
Methadone	N07BC02	109 (1.3)	
Piperacillin and tazobactam	J01CR05	102 (1.2)	
Midazolam	N05CD08	97 (1.2)	
Hydrochlorothiazide	C03AA03		53 (1.4)
Spironolactone	C03DA01		28 (0.7)
Pyrimethamine	P01BD01		13 (0.3)
Sulfadiazine	J01EC02		13 (0.3)
Dipyrone	N02BB02		8 (0.2)
Other		1,140 (13.6)	8 (0.2)
Total		8,372 (100.0)	3,790 (100.0)

ATC - Anatomical Therapeutic Chemical. Results expressed as n (%).

**Figure 1:**
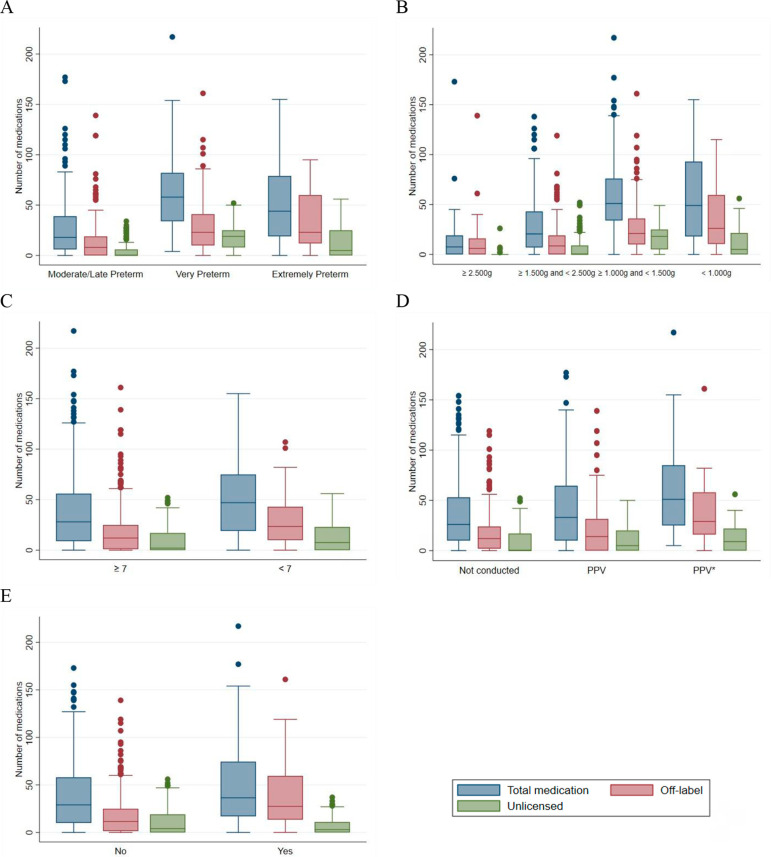
Boxplot of the relationships of the explanatory variables of interest with the number of drugs used (total, off-label and unlicensed) by gestational age (A); birth weight (B); 5-minute Apgar score (C); resuscitation maneuvers in the delivery room (D) and death (E). PPV - positive-pressure ventilation.

Bivariate analysis revealed statistically significant associations between the number of medications used (total, off-label and unlicensed) and the explanatory variables of interest. The very preterm and extremely preterm groups, with lower birth weights and 5-minute Apgar scores less than 7, who required advanced resuscitation maneuvers in the delivery room and who died required a higher median of products, considering both the total number of drugs and off-label drugs. Unlicensed medications were mostly used by very preterm newborns, with birth weights between 1,000g and less than 1,500g and 5-minute Apgar scores less than 7 (Table 4 and Figure 1).

**Table 4:** Relationship of the explanatory variables of interest with the number of drugs used (total, off-label and unlicensed)

	n	Median	Minimum value - maximum value	p value
Variables per total drug use				
Gestational age				< 0.001*
Moderate/late preterm	237	18.0	0 - 177	
Very preterm	117	58.0	4 - 217	
Extremely preterm	46	44.0	0 - 155	
Birth weight (g)				< 0.001*
≥ 2,500	48	7.5	0 - 173	
≥ 1,500 and < 2,500	178	20.5	0 - 138	
≥ 1,000 and < 1,500	122	51.0	0 - 217	
< 1,000	52	49.0	0 - 155	
5-minute Apgar score				0.003^†^
≥ 7	335	28.0	0 - 217	
< 7	54	47.0	0 - 155	
Resuscitation maneuvers in the delivery room				0.004*
No	217	26.0	0 - 154	
Only PPV §	156	33.0	0 - 177	
Advanced maneuver	27	51.0	5 - 217	
Death				0.030^†^
Yes	64	36.5	0 - 217	
No	336	29.0	0 - 173	
Variables by off-label drugs				
Gestational age				< 0.001*
Moderate/late preterm	237	8.0	0 - 139	
Very preterm	117	23.0	0 - 161	
Extremely preterm	46	23.0	0 - 95	
Birth weight (g)				< 0.001*
≥ 2,500	48	6.0	0 - 139	
≥ 1,500 and < 2,500	178	8.5	0 - 119	
≥ 1,000 and < 1,500	122	21.0	0 - 161	
< 1,000	52	26.0	0 - 115	
5-minute Apgar score				< 0.001^†^
≥ 7	335	12.0	0 - 161	
< 7	54	23.5	0 - 107	
Resuscitation maneuvers in the delivery room				< 0.001*
No	217	12.0	0 - 119	
Only PPV	156	14.0	0 - 139	
Advanced maneuvers	27	29.0	0 - 161	
Death				< 0.001^†^
Yes	64	27.5	0 - 161	
No	336	11.5	0 - 139	
Variables for unlicensed drugs				
Gestational age				< 0.001*
Moderate/late preterm	237	0	0 - 34	
Very preterm	117	19.0	0 - 52	
Extremely preterm	46	5.0	0 - 56	
Birth weight (g)				< 0.001*
≥ 2,500	48	0	0 - 26	
≥ 1,500 and < 2,500	178	0	0 - 52	
≥ 1,000 and < 1,500	122	18.0	0 - 49	
< 1,000	52	5.0	0 - 56	
5-minute Apgar score				0.020^†^
≥ 7	335	2.0	0 - 52	
< 7	54	7.5	0 - 56	
Resuscitation maneuvers in the delivery room				0.050*
No	217	0	0 - 52	
Only PPV	156	5.0	0 - 50	
Advanced maneuvers	27	9.0	0 - 56	
Death				0.820^†^
Yes	64	3.0	0 - 37	
No	336	4.0	0 - 56	

PPV - positive-pressure ventilation.

* p value estimated by the Kruskal-Wallis test;

† p value estimated by the Mann-Whitney test.

## DISCUSSION

The results of this study showed a high prevalence of off-label and unlicensed medication use among preterm infants admitted to neonatal ICUs in a rural region in the State of Bahia. The use of a greater number of these medications was associated with greater vulnerability of these individuals, who had lower gestational age and birth weight, in addition to an Apgar score less than 7. A higher number of off-label drugs was also associated with advanced resuscitation maneuvers in the delivery room and progression to death.

The prevalence rates for the use of off-label (79.0%) and unlicensed (55.5%) medications was higher than those reported in national and international studies. A cohort study published in Spain in 2019, conducted with newborns admitted to neonatal ICUs, found that 57.1% of patients received at least 1 off-label drug, while 32.1% received at least 1 unlicensed drug.^(16)^ Conversely, in an Italian study conducted in 2010 with newborns in neonatal ICUs, of the 88 treatments offered, 54.0% were off-label or unlicensed.^(5)^ In 2015, a study conducted in the Slovak Republic with newborns showed that out of 962 prescribed medications, 43% were classified as off-label and 4.8% as unlicensed.^(17)^

In a national cohort lasting 1 year in a neonatal ICU with 220 newborns, 49.3% of prescriptions were off-label, and 24.6% were unlicensed.^(18)^ Gonçalves et al.^(19)^ studied newborns admitted to the neonatal ICU of a university hospital in Brazil and found that 95.5% of newborns were prescribed at least 1 off-label drug and that 30.6% received at least 1 unlicensed drug. Notably, most of the available studies on this subject include neonates of all gestational ages admitted to the neonatal ICU, which differs from the methodology used in this cohort, in which only preterm infants were selected.

The literature shows that the use of off-label and unlicensed drugs is very common in pediatrics, with a high prevalence in neonatal ICUs.^(5,19,20)^ Some of these drugs have well-established use in protocols, clinical trials and meta-analyses but have not been investigated in controlled clinical trials that meet FDA criteria.^(21)^ The severity of the condition of neonates and the need for intensive care may explain the prescription of these drugs because these individuals are usually prescribed a large number of medications per day.^(22)^

In the present study, the off-label pharmaceuticals most commonly prescribed to preterm infants were systemic anti-infectives (63.8%), especially gentamicin and ampicillin, corroborating the findings of other studies.^(5,17-19,21,23,24)^ Gentamicin, despite being approved for use in newborns, according to the product specifications, should be administered every 8 hours.^(25)^ However, in local protocols, which are based on specific pediatric references,^(26)^ this drug is administered according to birth weight and can be used every 24, 36 or 48 hours. The use of ampicillin is approved only from 1 year of age, but there are pediatric clinical protocols that recommend its use in neonates, both for prophylactic and therapeutic purposes.^(26)^ A study conducted in Saudi Arabia in 2018 found that the most frequently used off-label antimicrobial was the combination of piperacillin and tazobactam.^(24)^ In another study conducted in Italy, the most commonly used off-label drugs were gentamicin, cefixime, ceftriaxone, piperacillin and amoxicillin.^(6)^

The heterogeneity of the use of antibacterial subgroups used in neonatal ICUs in different countries reflects the lack of a consensus based on clinical trials for the treatment and prophylaxis of the most prevalent neonatal infections, such as sepsis. Thus, this selection depends on the clinical experience and prescription policy of each hospital.^(23)^

The present study and others have shown that fluconazole, fentanyl, domperidone and ranitidine are widely used off-label drugs.^(16,17,21,23,27)^ Fluconazole was approved by the European Medicines Agency (EMA) for term newborns. This drug is also routinely used for antifungal prophylaxis in preterm infants, with controversial efficacy in the prevention of disseminated candidiasis. However, scientific societies have supported its use for preterm infants below 1,000g admitted to neonatal ICUs with a high prevalence of fungal infections.^(28)^

Fentanyl is among the most prescribed opioids in neonatal ICUs as it is well-established that pain is a factor that contributes to increased morbidity and mortality in neonates. However, according to the Institute for Safe Medication Practices (ISMP), this pharmaceutical belongs to the maximum alert group because, if administered incorrectly, it can cause serious health risks.^(29)^

Ranitidine is used for the treatment of gastroesophageal reflux disease, but its safety and efficacy are not well established for newborns, and its use is associated with longer hospitalization, necrotizing enterocolitis and death.^(30,31)^

Caffeine is a widely used drug in preterm infants admitted to neonatal ICUs and is the treatment of choice for apnea of prematurity. It reduces the need for mechanical ventilation and bronchopulmonary dysplasia and improves neurodevelopmental outcomes. In this study, caffeine was the most frequently used unlicensed drug, corroborating data from other studies.^(16,17,21,23)^ It was used in formulations manipulated by the surveyed units, as the hospitals did not have the licensed product available on the market, possibly due to its higher cost.

Dipyrone, also found among unlicensed drugs prescribed to preterm infants, is an analgesic not approved by the FDA because of the risk of inducing aplastic anemia and agranulocytosis^(21)^ and because there are no pharmacological and clinical studies on its use in neonates.^(32)^ Paracetamol is the only safe analgesic for neonates, but it is available only for oral use, which hinders its use in patients with contraindications to this administration route, and the onset of action is slow and of little effect in intense painful processes.^(32)^

The association between the increased use of off-label drugs and lower gestational age and birth weight is consistent with the finding from other international and national studies. Sucasas Alonso et al.^(16)^ found that the use of these products occurred in all preterm infants with a gestational age < 32 weeks. A similar result was found by Costa et al.,^(18)^ for whom 100% of preterm infants with extremely low birth weight were exposed to at least 1 off-label drug and 75.5% of the neonates in this classification were administered unlicensed medications.^(18)^ In Brazil, the frequency of use of at least 1 off-label drug for preterm infants with gestational ages less than 28 weeks and 32 weeks was 100% and 92.9%, respectively.^(19)^ Other studies did not find the same results.^(5,24)^

In Finland, a study conducted in a pediatric hospital also found that the chances of using off-label drugs increased significantly with the lower birth weight of the neonate, while a higher birth weight decreased the use of these drugs.^(33)^

Blumer and Reed^(34)^ argue that the physiological immaturity of preterm infants affects the absorption and distribution of drugs due to the composition of body compartments and to hemodynamic and metabolic factors. In addition, polypharmacy predisposes patients to greater pharmaceutical incompatibility or interaction between products and, consequently, brings greater risks of intoxication or adverse effects of drugs.

A Canadian cohort study conducted with preterm infants with a gestational age less than 31 weeks showed an association of better 5-minute Apgar scores with early use (less than 3 days of life) of caffeine, a drug considered unlicensed.^(35)^ In this study, it was found that the lowest 5-minute Apgar scores were associated with the greater use of off-label and unlicensed drugs, a finding that is probably related to neonates with fetal distress or neonatal asphyxia and who consequently had a greater need for the use of drugs.

In this cohort, preterm infants who required resuscitation in the delivery room, using positive-pressure ventilation alone or combined with cardiac massage and/or drugs, demanded a broader use of medications in general, including off-label and unlicensed drugs during the neonatal ICU stay. This finding is probably related to greater clinical instability and vulnerability of these preterm infants. A positive association was also demonstrated between the use of off-label drugs and death, a result observed by Carvalho et al.,^(21)^ who identified an association between the use of off-label and unlicensed prescriptions for patients with high median Neonatal Therapeutic Intervention Scoring System (NTISS) severity scores, i.e., patients whose condition was more severe.

Among the limitations of this study, there is possible information bias due to difficulty in obtaining data related to maternal characteristics, pregnancy, and the time of delivery in the medical records. Thus, we prioritized information on the health conditions of preterm infants and the neonatal care received, as these are more solid and reliable data obtained from medical records.

During the study period, the evaluated units did not perform calculations of any severity scores, such as the NTISS. Despite the possibility of its retrospective estimation, some necessary information was not obtained from the medical records, making it difficult to make comparisons between the population characteristics of different ICUs and in the service itself over time.

## CONCLUSION

The current study is unprecedented in the region and describes the characteristics of preterm infants admitted to neonatal intensive care units exposed to the use of medications. The prevalence of neonates exposed to off-label or unlicensed drugs during hospitalization was high and was associated with earlier prematurity, lower weights, lower 5-minute Apgar scores, receiving neonatal resuscitation and death. Antibiotics and caffeine were the most used off-label and unlicensed drugs, respectively. High exposure of neonates to off-label or unlicensed drugs in neonatal units is worrisome. Therefore, more studies are needed to achieve greater safety and quality of drug therapy used in neonatology.
